# Physico-Chemical, Mechanical, and Biological Properties of Polylactide/*Portulaca oleracea* Extract Electrospun Fibers

**DOI:** 10.3390/membranes13030298

**Published:** 2023-03-02

**Authors:** Nikoleta Stoyanova, Mariya Spasova, Nevena Manolova, Iliya Rashkov, Sabina Taneva, Svetlana Momchilova, Ani Georgieva

**Affiliations:** 1 Laboratory of Bioactive Polymers, Institute of Polymers, Bulgarian Academy of Sciences, Acad. G. Bonchev Street, bl. 103, BG-1113 Sofia, Bulgaria; 2 Department of Lipid Chemistry, Institute of Organic Chemistry with Centre of Phytochemistry, Bulgarian Academy of Sciences, Acad. G. Bonchev Street, bl. 9, BG-1113 Sofia, Bulgaria; 3 Institute of Experimental Morphology, Pathology and Anthropology with Museum, Bulgarian Academy of Sciences, Acad. G. Bonchev Street, bl. 25, BG-1113 Sofia, Bulgaria

**Keywords:** plant extract, *Portulaca oleracea*, polyester, electrospinning, normal fibroblasts, wound healing

## Abstract

Electrospinning was used to create fibrous polylactide (PLA) materials loaded with *Portulaca oleracea* (*P. oleracea)* plant extract obtained by supercritical carbon dioxide. Morphological, physico-chemical, mechanical, and biological characteristics of the fibers were studied. According to the SEM results, the diameters of smooth and defect-free fibers fabricated by a one-pot electrospinning method were at micron scale. All the obtained materials possess good mechanical properties. Additionally, it was found that the composite fibers exhibited considerable antioxidant activity. The antimicrobial activity of the fibrous materials against Gram-positive and Gram-negative bacteria was determined as well. In vitro studies showed that the electrospun biomaterials had no cytotoxic effects and that the combination of PLA and the *P. oleracea* extract in the fiber structure promoted cell survival and proliferation of normal mouse fibroblasts. The obtained results reveal that microfibrous mats containing the polyester—PLA and the plant extract—*P. oleracea* can be suitable for applications in wound healing.

## 1. Introduction

*Portulaca oleracea* (known as purslane) is a herbaceous annual that is distributed all over the world [1]. Purslane is grown as a specialty crop recognized for its dietary and therapeutic benefits, especially in Asia and in Mediterranean countries [2]. This herb is a rich source of essential nutrients, mainly minerals [3,4], vitamins A, C, E, and B, and omega-3 fatty acids [5,6], and contains bioactive phytochemicals such as carotenoids and phenolic antioxidants with proven health benefits [7,8]. Various parts of this herb possess antioxidant, anti-inflammatory, antitumor, antidiabetic, hepatoprotective, anti-insomnia, analgesic, skeletal muscle-relaxant, gastroprotective, neuroprotective, wound-healing, and antiseptic properties [9,10,11,12].

Biodegradable polymers (synthetic or natural) have attracted considerable interest in recent years. They find diverse applications in packaging, agriculture, medicine, etc. [13]. Due to their significant diversity and synthetic versatility, aliphatic polyesters are the most extensively investigated class of biodegradable polymers [14]. Polylactide (PLA) is a polymer derived from renewable resources that belongs to the class of biodegradable aliphatic polyesters. This polymer and its copolymers are extensively used as advanced drug delivery carriers [15].

In recent years, the electrospinning method has gained much attention due to the fact that it allows facile fabrication of continuous fibers with diameters ranging from tens of nanometers to several micrometers. This electrohydrodynamic process uses the application of a high voltage to generate a jet from polymer solution or melt which is highly stretched and elongated to generate nano- and microfibers [16]. Because of their beneficial characteristics such as a large surface-area-to-volume ratio and a high porosity with small pore size, the electrospun fibrous materials find applications in drug delivery [17], tissue engineering [18], cosmetics [19], filtration [20], protective clothing [21], food packaging [22], etc. The use of electrospun nano- and microfibers in the pharmaceutical industry has increased recently [23]. Antibiotics [24], proteins [25], extracts [26], DNA [27], RNA, and anticancer drugs [28,29], along with other bioactive substances [30], have been loaded in electrospun nanofibers to treat diverse diseases.

The preparation of PLA-based nanofibrous materials by the electrospinning technique has received growing attention [31]. The morphology of the PLA mats and their properties are affected by a variety of factors such as viscosity, conductivity, polymer concentration, surface tension, and solvent system [32]. Electrospun PLA nano- and microfibers possess biodegradability, biocompatibility, non-toxicity, and good thermo-mechanical properties, combined with high specific surface area, and therefore have been widely investigated for biomedical applications as wound dressings [33], drug carriers [34], and tissue engineering scaffolds [35], as well as membranes for separation [36]. However, studies reporting the incorporation of plant extracts in the PLA-based electrospun fibrous materials are scarce.

Plant species have been used medicinally since ancient times because of the better patient tolerance and acceptance. There are four main natural product sources: plants, animals, sea species, and microbes [37]. They show a remarkable variety in chemical composition and structure. Electrospinning can increase the therapeutic potential of plant extracts by encapsulating them in suitable polymer matrixes and in this way improving their bioavailability and maintaining the needed concentration of bioactive compound at the target area [38].

To our knowledge, there is only one study in the literature, which was carried out by us, reporting the incorporation of the *P. oleracea* plant extract in electrospun polymer fibers. Recently, we have performed experiments to find the optimal process conditions for the encapsulation of the *P. oleracea* extract in a suitable polymer matrix [39]. The effect of the extract concentration on the morphology and some properties of the obtained materials were determined. The novelty of the present study consists in revealing the physico-chemical, thermal, mechanical, and biological properties of recently developed PLA/*P. oleracea* fibrous materials, and in studying their potential for their application in the biomedical field.

## 2. Materials and Methods

### 2.1. Used Materials

The following materials have been used in the present study without further purification: PLA (Ingeo™ Biopolymer 4032D, NatureWorks, Minnetonka, MN, USA; Mw = 259,000 g mol^−1^, Mw/Mn = 1.94, as determined using size-exclusion chromatography with polystyrene standards), methylene chloride (DCM; Merck, Darmstadt, Germany), and ethanol absolute (Merck, Darmstadt, Germany).

Purslane plant material was collected in the Novo Zhelezare area (Plovdiv region, Bulgaria). Using a SEPAREX (France) high-pressure extractor with a 2 L extraction vessel and operating at a pressure of up to 1000 bar, 800 g of air-dried plant leaves and stems were milled and extracted with supercritical CO_2_. The supercritical extraction was conducted for 2 h at a pressure of 400 bar and a temperature of 80 °C.

DPPH (2,2-diphenyl-1-picrylhydrazyl) and a reference mixture of fatty acid methyl esters from Sigma-Aldrich (Darmstadt, Germany), ethidium bromide (EtBr; Sigma Chemical, Balcatta, WA, Australia), and acridine orange (AO; Sigma Chemical, Balcatta, WA, Australia) were used without additional purification as they were of analytical grade. Penicillin and streptomycin (LONZA, Cologne, Germany) antibiotics and fetal calf serum (FCS; Gibco, Wien, Austria) were added to Dulbecco Modified Eagle’s Medium (DMEM; Sigma-Aldrich, Darmstadt, Germany).

The mouse BALB/3T3 clone A31 cell line (ATCC, CCL-163) was obtained from the American Type Cultures Collection (ATCC, Rockville, MD, USA).

*Staphylococcus aureus* (*S. aureus*) 749 and *Escherichia coli* 3588 (*E. coli*) 74 were purchased from the National Bank for Industrial Microorganisms and Cell Cultures (NBIMCC), Sofia, Bulgaria.

### 2.2. Electrospinning for Preparation of Fibrous Mats

Fibrous PLA and PLA/*P. oleracea* materials were obtained by electrospinning, as described in detail elsewhere [39]. In brief, prior to electrospinning, the following solutions were prepared: (i) PLA (10 wt%) and (ii) PLA (10 wt%)/*P. oleracea* (7.5 wt% with respect to PLA weight). A mixed solution of DCM and EtOH (90/10 *w*/*w*) was used for the dissolution of the polymer and the extract.

The obtained PLA or PLA/*P. oleracea* solutions were put in a syringe (5 mL) fitted with a metal needle (gauge size: 20GX1½″) connected to the positively charged electrode of a high-voltage power supply (up to 30 kV). A grounded drum with a diameter of 45 mm was positioned 15 cm away from the needle tip, rotating at a constant speed of 1000 rpm. An infusion pump (NE-300 Just InfusionTM syringe pump, New Era Pump Systems Inc., Farmingdale, NY, USA) was used to supply the spinning solution at a regulated feed rate of 3 mL/h at a constant applied voltage of 25 kV, at a room temperature of 21 °C, and at a relative humidity of 52%.

### 2.3. Characterization of the Materials

The spinning solutions’ dynamic viscosity measurements were performed via a Brookfield DV-II+ Pro programmable viscometer equipped with a sample thermostatic cup and a cone spindle for the one/plate option, at room temperature (25 °C).

Scanning electron microscopy (SEM) was used to evaluate the fibers’ morphology. Before the sample observation, the fibrous materials were vacuum-coated with a fine gold layer and analyzed using a Jeol JSM-5510 scanning electron microscope (JEOL Co., Ltd., Tokyo, Japan).

Using the ImageJ software [40], at least 50 fibers from SEM micrographs were evaluated in order to determine the average fiber diameter, fiber distribution, and morphology according to the previously reported criteria for the complex evaluation of electrospun mats [41].

Surface wettability analysis was used in order to evaluate the static contact angle via a DSA 10-MK2 drop shape analyzer system (Krüss, Hamburg, Germany) at 20 ± 0.2 °C. Contact angles of the fibrous materials were measured by dropping a deionized water droplet (10 μL) controlled by a computer dosing system on the surface. The droplet’s temporal photographs were captured. Computer analysis of the obtained pictures was used in order to determine the contact angles. The final results represented an average of 20 measurements taken on various regions of the mat surfaces.

Thermogravimetric analysis (TGA) was carried out on the Perkin Elmer TGA 4000 (Waltham, MA, USA) at a 10 °C/min heating rate under argon at a flow of 60 mL/min. Pyris v. 11.0.0.0449 software was used for instrument control, data collecting, and data processing.

The mechanical properties of the fibrous materials were determined via tensile measurements performed in a single column system for mechanical testing, the INSTRON 3344, equipped with a loading cell of 50 N and the Bluehill universal software. The initial length between the clamps was 40 mm and the used stretching rate was 10 mm/min. The fibrous samples were cut in the direction of the collector rotation with dimensions of 20 × 60 mm^2^. A Digital Thickness Gauge FD 50 (Kafer GmbH, München, Germany) was used to determine the thickness of the fibrous materials. The average thickness was ca. 300 μm ± 20 nm. For the sake of statistical significance, 10 specimens of each sample were tested, after which the average values of Young’s modulus, the ultimate stress, and maximum deformation at break were determined.

The antioxidative properties of the materials was evaluated using the 2,2-diphenyl-1-picryl-hydrazyl-hydrate (DPPH) free radical method. A volume of 0.5 mL of an ethanol solution of *P. oleracea* (0.375 mg) was mixed with 2.5 mL of an ethanol solution of DPPH at a concentration of 1 × 10^4^ mol L^−1^. Three milliliters of DPPH solution in ethanol was added to PLA (5 mg mat) or PLA/*P. oleracea* (5 mg mat containing 0.375 mg of *P. oleracea*) fibrous mats. The as-prepared blended solutions were incubated in the dark for half an hour at room temperature (21 °C). Using a DU 800 UV-visible spectrophotometer (Beckman Coulter, Brea, CA, USA), the amount of DPPH radicals left in the solution was determined. The following equation was used to assess the antioxidant activity (AA):

(1) $$\mathrm{Inhibition},\;\mathrm{AA},\%=\left[\frac{(A_{\mathrm{DPPH}}-A_{\mathrm{sample}})}{A_{\mathrm{DPPH}}}\right]\;\times \;100\;,$$

where A_sample_ is the absorbance for the DPPH• solution at 517 nm after the addition of the solution containing plant extract or fibrous materials, and A_DPPH_ is the absorbance for the DPPH• solution at 517 nm. Experiments were performed in triplicate.

### 2.4. Determination of Fatty Acids Composition and Acid Value

Gas chromatography (GC) with a flame ionization detector was used for the determination of fatty acids composition after acid-catalyzed transesterification of plant supercritical CO_2_ extract to methyl esters [42]. Prior to GC analysis, fatty acid methyl esters (FAME) were purified by preparative thin-layer chromatography on a silica gel plate using a mobile phase of hexane-acetone (100:6 *v*/*v*). GC was conducted on a Shimadzu GC 2030 chromatograph equipped with a flame ionization detector and a Simplicity Wax capillary column (30 m × 0.32 mm × 0.25 μm, Supelco). The operating temperature program was a gradient from 170 °C to 260 °C at 2 °C/min and 5 min held at the final temperature. The injector and detector temperatures were 260 °C and 280 °C, respectively. Nitrogen was used as a carrier gas at 0.6 mL/min flow rate, with a split ratio of 1:50. Peak identification was done according to retention times and compared to that of a standard FAME mixture. The acid value (AV) was estimated by titration with ethanolic KOH.

### 2.5. Antibacterial Activity Assessment

The antibacterial activities of the electrospun mats were determined against the Gram-positive bacteria *S. aureus* 749 and Gram-negative bacteria *E. coli* 3588 by applying the disk diffusion assay. For that purpose, in vitro studies were performed using the Tryptone glucose extract agar (DIFCO Laboratories, Detroit, MI, USA) solid medium. The surface of the solid agar was inoculated with a suspension of cell culture with a cell concentration of 1 × 10^5^ cells/mL. Within 5–10 min after inoculation, PLA mat and PLA/*P. oleracea* were placed on the inoculated surface (one disc with a diameter of 17 mm and weight of 5.0 mg per Petri dish). The Petri dishes were incubated for 24 h at 37 °C. Subsequently, the diameters of the inhibition zones around the disks were observed.

### 2.6. Dual Staining Using AO and EtBr

Dual staining of mouse BALB/c 3T3 fibroblast cells was performed using acridine-orange (AO) and ethidium bromide (EtBr) in order to evaluate cell death. The cells were plated at a concentration of 2 × 10^5^ cells × mL^−1^ Dulbecco’s Modified Eagle Medium (DMEM) supplemented with 10% fetal bovine serum (FBS) on glass lamellas, placed at the bottom of 24-well plates, and incubated at 37 °C for 24 h in a CO_2_ incubator to form a monolayer. PLA and PLA/*P. oleracea* fibrous mats were then sterilized using UV light and placed in the 24-well plates for additional 24 h of incubation. After that, the electrospun mats were removed and glass lamellas were washed twice with phosphate-buffered saline (PBS, pH 7.4) to remove unattached cells. Subsequently, the lamellas were stained with AO and EtBr at a ratio of 1:1 (10 μg/mL), and were observed on a fluorescence microscope (Leika DM 5000B, Wetzlar, Germany).

### 2.7. Mouse Fibroblast Adhesion on the Fibrous Surface

The adhesion of mouse BALB/3T3 cells on the surface of PLA and a PLA/*P. oleracea* fibrous mat was monitored by direct SEM observation. For that purpose, the fibrous samples were placed in 24-well tissue culture plates (Falcon Becton Dickinson, USA). A total of 2 × 10^5^ cells per well were seeded on each sample and cultured for 72 h in 1 mL DMEM with 10% FBS. In order to prevent the mats from floating, thin Teflon rings adapted to the inner diameter of the wells were used. The samples were fixed after 72 h of incubation with 2.5 wt% glutaraldehyde solution in saline at 4 °C for 4 h. Then, the samples were carefully washed three times with saline and, prior to freeze-drying, with distilled water. Before the SEM observations, the specimens were vacuum-coated with gold under vacuum.

## 3. Results and Discussion

### 3.1. Morphology and Physico-Chemical Properties of the Fibrous Mats

**Figure 1:**
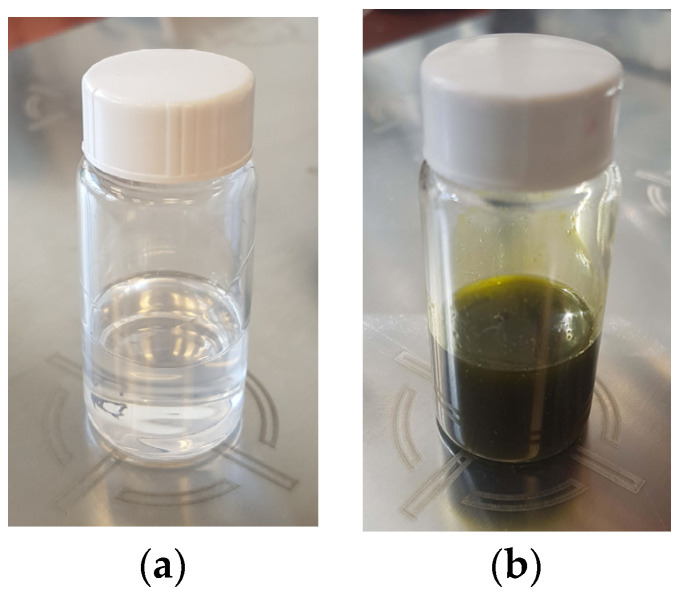
Digital images of spinning solutions of: (**a**) PLA and (**b**) PLA/*P. oleracea*.

In the present study, the morphology and properties of fibrous materials based on biocompatible and biodegradable polyester—PLLA and a natural plant extract of *P. oleracea* prepared by one-pot electrospinning were investigated. Electrospinning is a versatile technique for the fabrication of nanofibrous materials and the final morphology strongly depends on the intrinsic properties of the solution itself, its viscosity, and conductivity. It is known that electrospinning of low-viscosity solutions results in discontinuous fiber formation. Therefore, the dynamic viscosities of PLA and PLA/*P. oleracea* spinning solutions were measured prior to conducting the one-pot electrospinning. The determined viscosity value of the PLA solution (10 wt%) was 1180 ± 10 cP. The addition of the crude extract to the PLA solution resulted in a change of the solution color from transparent to saturated green (Figure 1) and led to a significant increase of the measured value of the dynamic viscosity to 4350 ± 15 cP. We assume that some components of the *P. oleracea* extract restricted the possibility of the polymer chains to move which resulted in the significant increase of the mixed solution’s viscosity.

**Figure 2:**
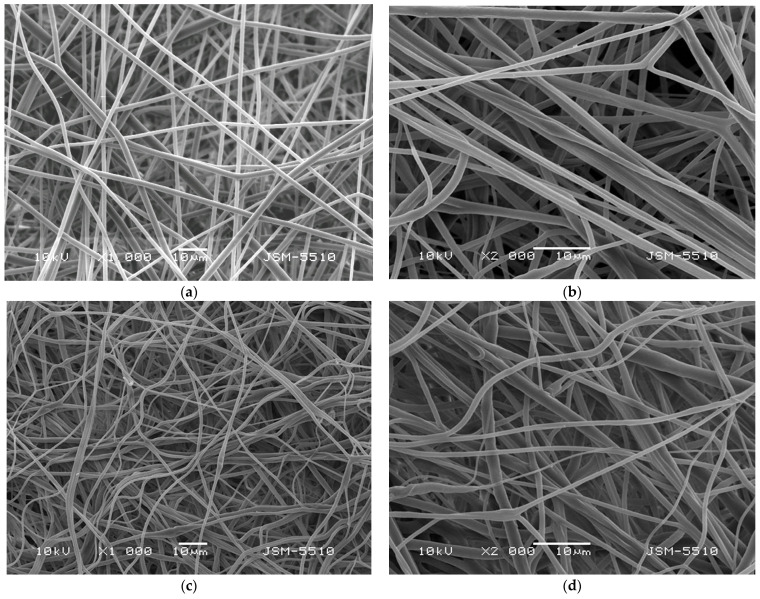
SEM images of: (**a**,**b**) PLA fibrous mat and (**c**,**d**) PLA/*P. oleracea* fibrous mat at magnifications ×1000 (**a**,**c**) and ×2000 (**b**,**d**).

Subsequently, after the spinning solutions preparation and measuring of their dynamic viscosities, the solutions were subjected to electrospinning. The morphology of the obtained electrospun fibrous materials was evaluated by using scanning electron microscopy. Representative SEM images of the PLA and PLA/*P. oleracea* mats were shown in Figure 2. The shown SEM micrographs at different magnifications reveal the morphology of the obtained fibrous materials. As can be easily seen, the electrospinning of the PLA solution with concentration 10 wt% reproducibly resulted in the fabrication of fibers with mean fiber diameter of 1100 ± 200 nm. The prepared PLA fibers are continuous, defect-free, and with smooth surface. The addition of the plant extract in the PLA spinning solution and its consequent subjection to electrospinning resulted in the fabrication of fibers with larger diameters compared to PLA alone. The mean diameter of the PLA/*P. oleracea* fibers was 2200 ± 550 nm. The detected increase in the diameters of the composite fibers is most probably due to the significant increase in the dynamic viscosity of the PLA/*P. oleracea* spinning solution (4350 ± 15 cP) compared to the viscosity value of the PLA solution (1180 cP).

The wettability of electrospun fibers is one of the most important factors for their applications, especially in biomedicine, pharmacy, agriculture, etc. Therefore, the surface wettability of the produced fibrous materials was assessed. The results from the water contact angle measurements showed that the PLA mats were hydrophobic since a water drop placed on them maintained its spherical form at a water contact angle of 110 ± 3.5°. The presence of the extract in the fibers in the case of the PLA/*P. oleracea* mat resulted in a slight reduction in the water contact angle value. The 94 ± 2.5° contact angle value shows that the fabricated PLA/*P. oleracea* mats are hydrophobic as well.

**Figure 3:**
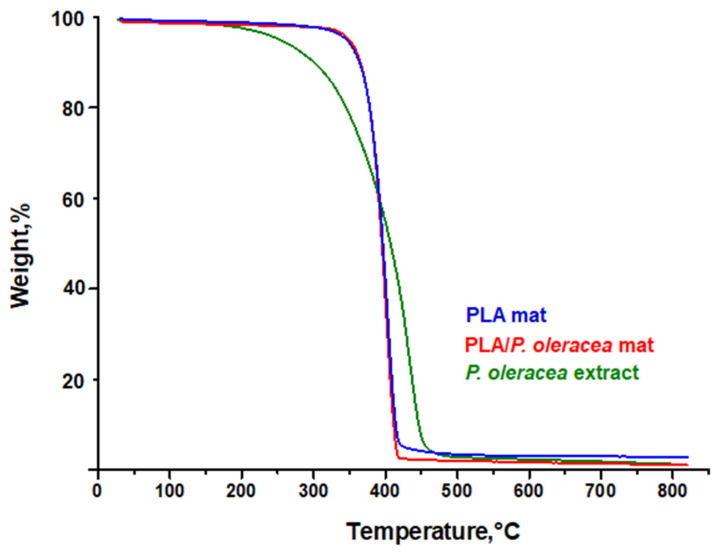
TG thermograms of crude *P. oleracea* extract, PLA, and PLA/*P. oleracea* mats.

The effect of the plant extract in the PLA matrix on the thermal characteristics of the hybrid fibrous mats was studied by thermogravimetric analyses. The thermograms of electrospun PLA materials and hybrid electrospun PLA/*P. oleracea* mats are shown in Figure 3. The TGA of the pristine natural plant extract of *P. oleracea* was performed as well. Thermal stability of the neat extract showed a continuous mass loss in a one-degradation step, being almost fully degraded at 800 °C. The weight loss (ca. 3–4%) started to occur at approximately 150 °C, which can be ascribed to the evaporation of moisture (desorption of water) or some extract volatiles. The main mass loss started at around 300 °C.

As seen in Figure 3, the electrospun PLA and PLA/*P. oleracea* mats showed one decomposition peak. The thermal decomposition of electrospun PLA mat started at 330 °C and ended at 425 °C due to the decomposition of the polyester. Additionally, the presence of the natural extract in a concentration of 7.5 wt% does not alter the thermal behavior of the composite mat. The thermal degradation of electrospun PLA/*P. oleracea* mats began at 325 °C and ended at 430 °C. The residual mass at 800 °C was 1.20%, 1.20%, and 2.83% for the neat extract, the PLA/*P. oleracea* mat, and the PLA mat, respectively.

**Figure 4:**
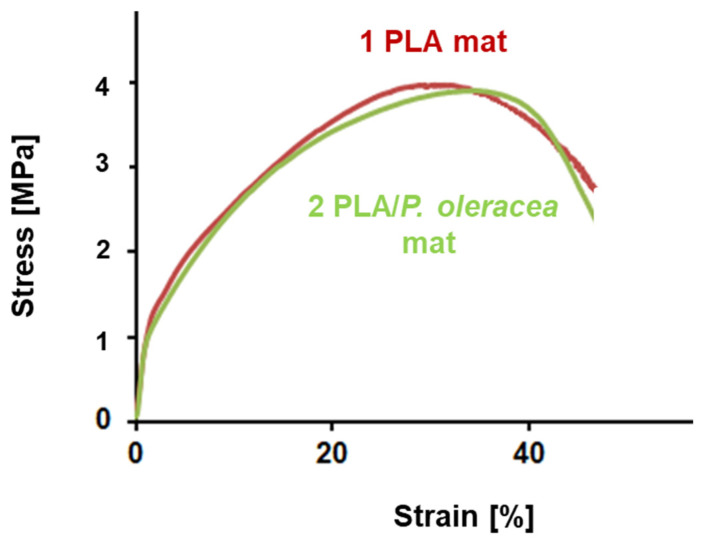
Stress–strain curves of: electrospun PLA mat and PLA/*P. oleracea* mat.

The mechanical properties are one of the most important properties of the electrospun fibers. They play an important role in determining the fibers’ applications. The mechanical characteristics of the fibrous mats depend strongly on measurement technique, conditions of fiber fabrication, fiber orientation, point bonding, crosslinking, etc. The addition of a second component to the spinning solution might have a significant effect on the mechanical behavior of the resulting composite fibers. Therefore, it is crucial to study the influence of the extract on the mechanical properties of the hybrid PLA/*P. oleracea* mats. The mechanical characteristics of the obtained electrospun mats were determined using a single-column tensile testing machine. The typical stress–strain curves of PLA and PLA/*P. oleracea* mats are shown in Figure 4. The tensile strength values of the PLA mat and the hybrid mat containing the natural extract were very similar. The tensile strength of the PLA/*P. oleracea* mat was ca. 3.78 MPa, while the tensile strength of the PLA fibrous material reaches 3.9 MPa. This finding proved that the incorporation of the *P. oleracea* extract (at a concentration of 7.5 wt% with respect to the polymer weight) in the polymer matrix does not lead to a decrease in the mechanical properties of the composite material, thus preserving its good mechanical properties.

Various parts of purslane are known for medicinal and pharmacological uses because of its antioxidant activity [9]. The antioxidant activity of plants is due to their antioxidants, the majority of which are phenolic compounds such as phenolic acids and flavonoids, along with organic acids such as rosmarinic, caffeic, chlorogenic, p-coumaric, ferulic acids, quercetin, rutin, kaempferol, fumaric, oxalic, citric, acotinic, and malic acids. These compounds are capable of reducing oxidative stress by scavenging free radical species, and many of them have been identified in *P. oleracea* [43]. There are no data in the literature about the composition of the supercritical CO_2_ *P. oleracea* extract. Our initial analyses revealed significant content of waxes, chlorophyll, about 40% lipids of neutral and polar classes in comparable amounts, some presence of terpenes, and other compounds. As for the fatty acids composition of this extract, lignoceric acid (24:0) was predominant at 30%, followed by behenic acid (22:0) at 18%, linoleic and linolenic acids (18:2 and 18:3, respectively) at 10%, palmitic (16:0), arachidic (20:0), and cerotic (26:0) acids at 7%, stearic (18:0) and palmitoleic (16:1) acids at 3%, oleic acid (9–18:1) at 2%, and other four acids (12:0, 14:0, 11–18:1 and 20:1) at below 1%. A high acid value (14 mg KOH/g) had been expected most probably because of the oxalic acid presence in such plants.

**Figure 5:**
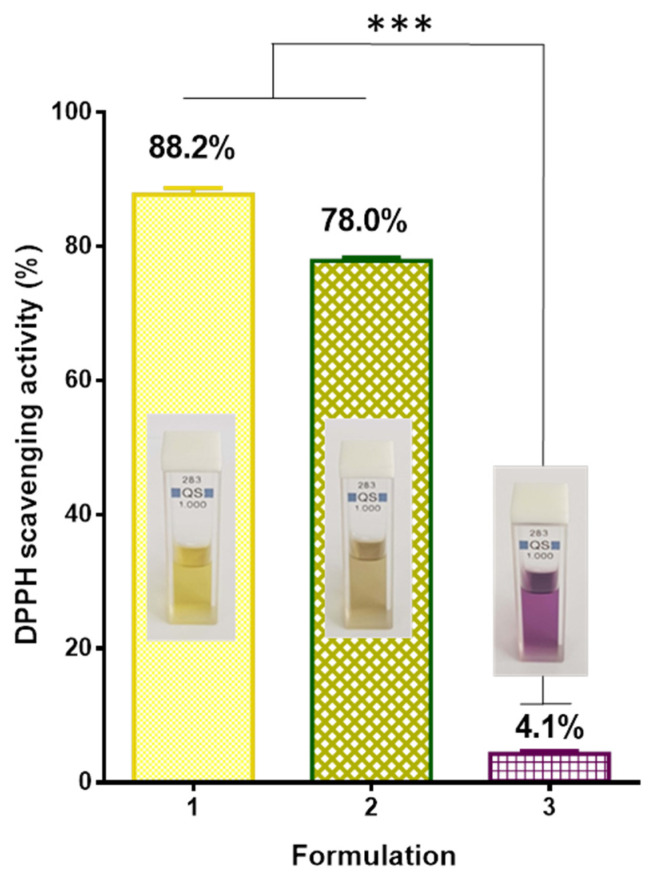
Antioxidant activity: 1—ethanol solution of the *P. oleracea* crude extract; 2—PLA/*P. oleracea* mat; 3—PLA mat and inset—digital images of 1—DPPH solution in the presence of the *P. oleracea* crude extract; 2—DPPH solution in the presence of a PLA/*P. oleracea* mat; and 3—DPPH solution in the presence of a PLA mat. *** *p* < 0.001.

The antioxidant activity of the fibrous mats obtained in the present study was evaluated using the DPPH radical scavenging assay. This method was used to screen the radical scavenging activity of the *P. oleracea* crude extract as well. It is known that DPPH creates a violet color in ethanol or methanol solution; however, in the presence of antioxidants, the color fades to yellowish hues. The results of the antioxidant activity as well as the digital images of the DPPH solution in the presence of different samples were presented in Figure 5. As it can be easily seen, the color of the DPPH solution in contact with the fibrous PLA mat was deep violet and the absorbance of the radical decreased by approximately 4.1%. In contrast, the color of the DPPH solution in contact with PLA/*P. oleracea* mat changed its color to yellowish and the DPPH absorbance decreased by approximately 78%, revealing high antioxidant activity of the extract-containing fibrous material. For the sake of comparison, the change in absorbance of the DPPH solution upon contact with an ethanol solution of *P. oleracea* was measured and it was 88.2%. The obtained results revealed that the incorporated *P. oleracea* extract preserves it strong antioxidant activity into the polymer fibrous mat and imparted antioxidant properties to the hybrid material.

### 3.2. Antibacterial Activity of the Fibrous Materials

**Figure 6:**
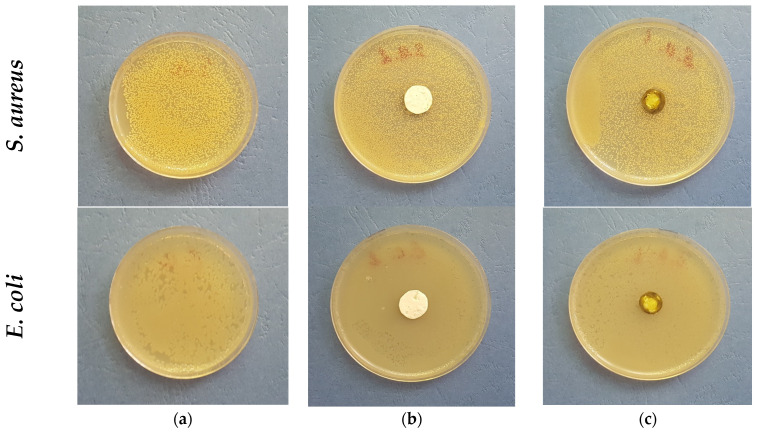
Digital photographs of (**a**) control bacteria; (**b**) PLA mat; and (**c**) PLA/*P. oleracea* mat after 24 h with *S. aureus* and *E. coli*. The cell type is marked in the left of each row.

There are few reports showing the antimicrobial potential of purslane [44]. It has been reported that the *Portulaca elatior* root contains a trehalose-binding lectin possessing antibacterial and antifungal activities [45]. Further, Du et al. reported that *Portulaca oleracea* L. exhibited different levels of antibacterial activities against Gram-positive and Gram-negative bacteria, showing antibacterial activity in Gram-positive bacteria [46]. The authors suggest that this might be due to the several structural differences between Gram-positive and Gram-negative bacterial cell walls, as the latter has an outer membrane and a unique periplasmic space. Knowing this literature data, it was of interest to us to determine the antibacterial potential of the electrospun fibrous mats obtained in this study. Thus, discs with diameters of 17 mm were cut and placed in contact for 24 h with Gram-positive bacteria—*S. aureus* and Gram-negative bacteria—*E. coli*. The digital images of the Petri dishes are shown in Figure 6. As seen in Figure 6a, the bacteria cells grow normally (control). As expected, the PLA fibrous mats do not show any antibacterial activity against the tested pathogenic bacteria. However, no zones of inhibition were detected around the fibrous PLA/*P. oleracea* discs either.

### 3.3. Cytotoxicity Assay and Cell Staining

**Figure 7:**
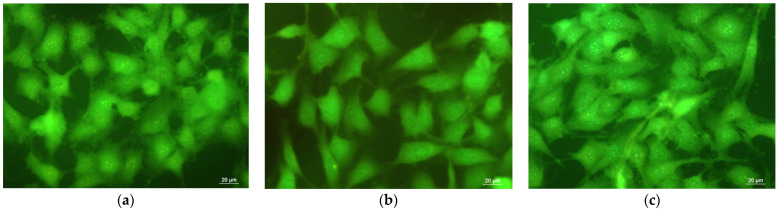
Fluorescence micrographs of AO and EtBr double-stained mouse BALB/c 3T3 fibroblast cells incubated for 24 h: (**a**) untreated cells; (**b**) PLA mat; and (**c**) PLA/*P. oleracea* mat. Live cells are shown in green. Bar: 20 μm.

Nano- and microfibrous scaffolds created by electrospinning exhibit multiscale functionalities, including the ability to release locally specific bioactive molecules from synthetic or natural origin to targeted cell types [47]. This feature is highly desirable in regulating appropriate cell phenotypes for tissue engineering and wound healing applications [48,49]. Furthermore, the electrospun mats resemble the extracellular matrix of human body tissues and facilitate tissue regeneration. *P. oleracea* extract possesses a wide spectrum of pharmacological properties due to the presence of active components such as flavonoids, terpenoids, and vitamins that contribute to epithelialization and promote skin renewal. The biocompatibility of the obtained materials is a main factor for their future biomedical applications. The in vitro compatibility of the PLA and PLA/*P. oleracea* mats was assessed by MTT assay [33], while the cellular morphology of mouse fibroblasts was observed on a fluorescence microscope after staining cells that were cultivated in contact with fibrous materials. Fibroblasts play a key role in restoring the integrity of injured tissue. Therefore, mouse BALB/c 3T3 fibroblasts were cultured for 24 h with electrospun PLA and PLA/*P. oleracea* mats, and were then stained using fluorescent dyes (AO and EtBr). This staining method allows to discriminate between dead and viable cells. Acridine orange stains both live and dead cells, emitting green fluorescence as a result of its intercalation in the double-stranded DNA. As opposed to AO, EtBr is not able to pass through the membrane of viable cells and stains only dead and late apoptotic cells with poor membrane integrity, generating red fluorescence. Figure 7 presents the fluorescence micrographs showing the cells’ morphology. Untreated fibroblast cells are characterized by a normal morphological structure with pale green nuclei and bright green nucleoli. No change was observed in the staining of the nuclei and cytoplasm in cells after their treatment with PLA and PLA/*P. oleracea* mats. The cell morphology remained normal. Furthermore, the previously obtained results from the MMT test as well as the cell staining reveal that the number of fibroblasts increased after being in contact with the fibrous mats containing the plant extract. This result shows that the electrospun PLA/*P. oleracea* mat is a prospective biomaterial with no toxicity supporting fibroblast attachment and proliferation in vitro.

**Figure 8:**
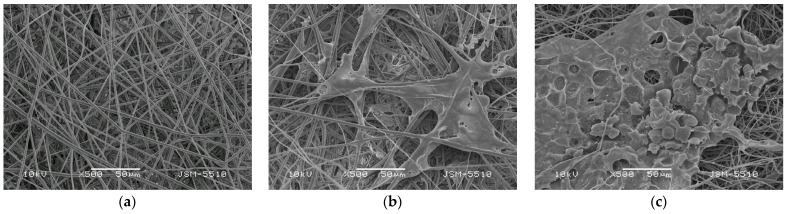
SEM micrographs of: (**a**) PLA fibers; (**b**) mouse fibroblasts on PLA fibers; and (**c**) mouse fibroblasts on PLA/*P. oleracea* after 72 h contact.

The mouse BALB/3T3 cell line is suitable for preliminary assessment of cell viability, proliferation, and adhesion to the obtained fibrous materials. The adhesion of fibroblasts to the surface of the prepared PLA and PLA/*P. oleracea* fibrous materials was assessed by SEM analysis. As shown in Figure 8, the fibroblasts attach well to all fibrous materials, particularly when the plant extract of *P. oleracea* is loaded into the PLA fibers (Figure 8c). Moreover, considerable cell spreading is observed. The cells gain a specific shape following the fibers and spreading between them. This result revealed that the mouse fibroblasts adhere and spread well on the fibrous PLA and PLA/*P. oleracea* mats. This finding confirms the cell compatibility of the created novel materials and their potential use for wound healing and tissue engineering applications.

## 4. Conclusions

In the present study, the physico-chemical, mechanical, and biological properties of electrospun PLA and PLA/*P. oleracea* materials were characterized. The incorporation of a crude *P. oleracea* extract, obtained by using supercritical CO_2_, imparted to the fibrous mat strong antioxidant activity while preserving the good mechanical properties of the fibers. Moreover, the in vitro tests with normal fibroblasts reveal that the electrospun PLA/*P. oleracea* material is biocompatible, promoting fibroblast adhesion, attachment, and proliferation. Thus, the created fibrous materials containing the plant extract could be suitable candidates for wound dressing applications.

## Data Availability

The data presented in this study are available on request from the corresponding author.
